# Supplementary material for: Comparison of Chemical and Sensory Profiles between Cabernet Sauvignon and Marselan Dry Red Wines in China
Source: Foods. 2023 Mar 5;12(5):1110. doi: 10.3390/foods12051110 (PMC10000944; doi:10.3390/foods12051110)
Supplement: Supplementary file 1 [file foods-12-01110-s001.zip › foods-2159621-supplementary.pdf]

Supplementary Material

Table S1 Oenological parameters of Cabernet Sauvignon and Marselan dry red wines from five producing regions in China.

NO	Label	Wineries	Alcohol (%vol)	Total sugar(g/L)	Total acidity(g/L)	pH
1	CS-1	Chateau Yunmo Greatwall	15.00	4.8	6.3	-
2	CS-2	Amethyst Manor	14.10	3.7	5.16	3.61
3	CS-3	Martin Vineyard	13.00	3.7	6	3.71
4	CS-4	Grace Vineyard	13.90	2.8	5.6	3.65
5	CS-5	Huangkou Winery	14.50	3.8	6.3	3.64
6	CS-6	Chateau Huahao	14.50	2	5.9	3.75
7	CS-7	Chateau Huahao	-	-	-	-
8	CS-8	Chateau Zhongfei	14.00	3.8	6	3.78
9	CS-9	Chateau Zhongfei	-	-	-	-
10	CS-10	Tiansai Vineyards	14.70	2.7	6.05	3.69
11	CS-11	Tiansai Vineyards	-	-	-	-
12	CS-12	CITIC Guoan Winery	13.40	4.8	5.5	3.7
13	MA-1	Chateau Yunmo Greatwall	-	-	-	-
14	MA-2	Amethyst Manor	13.50	3.5	5.64	3.57
15	MA-3	Martin Vineyard	13.50	4	5.2	3.85
16	MA-4	Grace Vineyard	14.00	2.9	2.9	3.5
17	MA-5	Huangkou Winery	14.80	4	6.2	3.6
18	MA-6	Chateau Huahao	14.90	2.2	5.8	3.78
19	MA-7	Chateau Huahao	-	-	-	-
20	MA-8	Chateau Zhongfei	13.80	3.5	6	3.77
21	MA-9	Chateau Zhongfei	-	-	-	-
22	MA-10	Tiansai Vineyards	15.10	3.4	5.83	3.57
23	MA-11	Tiansai Vineyards	-	-	-	-
24	MA-12	CITIC Guoan Winery	13.00	4.2	5.4	3.7

"-" means the oenological parameters were not collected from the wineries.

Table S2 Quantitative information of major aroma compounds by HS-SPME-GC-MS*.

NO	Retention index	Compound	Quantitative ion (m/z)	Calibration curve			Linear range (μg/L)
				slope	intercept	R^2	
1	719	Ethyl acetate	43	498.9	-3077.6	0.9989	3540.2-141608
2	1009	Ethyl butanoate	71	1152.2	-16.869	0.9995	20.52-1026
3	1044	Ethyl 2-methylbutanoate	52	412.56	-0.5746	0.9967	1.72-68.8
4	1054	Ethyl 3-methylbutanoate	88	575.72	-1.583	0.9966	3.52-140.8
5	1067	Isobutanol	43	50641	-5669.6	0.9988	4365.8-523896
6	1108	Isoamyl acetate	43	385.04	133.25	0.993	136.12-3403
7	1145	Isoamyl alcohol	45	1000000	-159.45	0.9992	2552.6-306312
8	1192	Ethyl hexanoate	55	463.42	78.977	0.997	173.44-3468.8
9	1222	Hexyl acetate	88	1.9344	1.3326	0.9979	1.92-38.4
10	1259	Ethyl heptanoate	43	8.492	-0.0616	0.9959	0.648-12.96
11	1320	Ethyl lactate	88	2000000	29038	0.9947	41200-247200
12	1326	1-Hexanol	45	20684	1.8352	0.999	94.8-1422
13	1345	(Z)-3-Hexenol	41	384.4	-3.5745	0.9987	5.6-224
14	1365	Methyl octanoate	67	2376.1	0.4117	0.9884	1.96-19.6
15	1376	Ethyl octanoate	74	3274.8	-226.43	0.9867	85.6-2054.4
16	1425	Acetic acid	88	0.0544	0.2496	0.9849	0.205-1.025
17	1432	1-Heptanol	43	14.471	-6.292	0.9913	1.04-20.8
18	1436	Isoamyl hexanoate	70	14.108	0.4649	0.9975	0.82-16.4
19	1446	Furfural	70	52.527	43.79	0.9971	23.66-709.8
20	1451	2-Ethylhexanol	96	313.94	-3.1501	0.9866	0.74-29.6
21	1471	Benzaldehyde	57	2062.8	-15.681	0.9964	0.68-54.4
22	1514	Ethyl nonanoate	77	2126.1	5.1344	0.9993	12.32-30.8
23	1522	Ethyl 2-hydroxy-4-	88	1786.6	-24.78	0.9992	38.24-573.6
24	1527	Linalool	69	44.26	0.4271	0.9948	0.264-13.2
25	1529	1-Octanol	71	1537.6	-12.451	0.9995	1.72-68.8
26	1538	Isobutyric acid	56	255.58	-1.177	0.99922	1.566-401
27	1549	2,3-Butanediol	43	20000000	71657	0.9964	88060-1320900
28	1568	5-Methylfurfural	45	88.31	-0.49	0.9989	2.164-554
29	1561	Terpinen-4-ol	110	190.56	-0.4983	0.9942	0.78-6.24
30	1588	Ethyl 2-furoate	71	1637.45	1.35	0.9618	3.611-462.25
31	1608	Ethyl decanoate	95	99.98	0.104	0.9928	4.192-134.14
32	1626	1-Nonanol	88	63433.41	45.12	0.9969	0.289-592
33	1641	Isoamyl octanoate	56	1569.95	-0.586	0.9993	0.498-63.69
34	1646	Isovaleric acid	70	161.11	170.63	0.9936	90.72-2268
35	1650	Ethyl succinate	60	1568.5	35.83	0.9996	47.6-1428
36	1661	α-Terpineol	101	4.9102	1.3078	0.9924	0.82-12.3
37	1681	Methionol	59	16985	136.54	0.9929	406.4-3251.2
38	1698	1-Decanol	106	1098	-6.2996	0.9989	1.6-64
39	1742	Methyl salicylate	70	3679.15	1.419	0.9988	0.33-168
40	1765	Ethyl benzeneacetate	120	463.06	-0.3661	0.9999	1.52-18.24
41	1771	β-Phenethyl acetate	91	677.46	5.3965	0.994	4.48-89.6

42	1801	β-Damascenone	104	176.15	3.24	0.9997	0.0996-102
43	1808	Ethyl dodecanoate	43	6873.7	-2.6653	0.9991	20.4-306
44	1828	Benzyl alcohol	71	0.00005	-0.0008	0.996	25.44-1526.4
45	1857	Phenylethyl Alcohol	52	3000000	-746.89	0.9997	4457.6-111440
46	1894	β- Ionone	88	4.1966x	0.4119	0.9965	0.44-7.04
47	1925	(Z)-Oaklactone	43	369.85	16.152	0.9907	19.44-116.64
48	1946	Phenol	43	451.12	-0.1458	0.9901	1.6-32
49	1984	Octanoic acid	45	111.54	0.336	0.9911	5.007-640.94
50	2046	Ethyl cinnamate	55	228.32	0.204	0.9981	12.03-385
51	2175	Ethyl hexadecanoate	43	315.96	-0.56	0.9997	0.418-107
52	2257	*n*-Decanoic acid	88	1065.4	-99.246	0.9839	60.96-1143

* A synthetic matrix (12% ethanol/water solution containing 2 g/L glucose and 5.3 g/L tartaric acid, pH = 3.5) was used to establish the calibration curves. The standard solution was diluted into fifteen levels. Aroma standards of each level were extracted and analyzed in triplicate under the same conditions as the samples.

Table S3 Quantitative information for aliphatic lactones by SPE-GC-QqQ-MS/MS*.

Compound	Ion1 (m/z)	Ion2 (m/z)	Calibration curves			Linear range (μg/L)
			Slope	intercept	R2	
Butyrolactone	86-42	-	9235.7	-2430.2	0.9926	172.422-44140
Pantolactone	130-71	71-71	317398	-29.468	0.9902	45.313-1450
γ-Hexalactone	114-85	114-55	5144.5	-3.2914	0.9938	2.188-560
γ-Octalactone	124-109	124-95	7698.4	-0.3032	0.9988	0.195-200
δ-Octalactone	99-71	114-71	12.194x	0.5866	0.9956	0.469-30
γ-Nonalactone	128-95	128-71	9.0372	-1.3347	0.9994	0.820-105
γ-Decalactone	128-95	128-71	143.05	-1.2665	0.9979	0.195-50
δ-Decalactone	152-97	99-71	21.76	-1.4659	0.998	0.8595-55
γ-Undecalactone	128-95	128-71	155.96	0.1883	0.9983	0.225-115
γ-Dodecalactone	128-95	128-71	216.97x	0.4381	0.9932	0.186-95
δ-Dodecalactone	114-73	99-71	24.998	0.7568	0.9881	0.234-60

* A synthetic matrix (12% ethanol/water solution containing 2 g/L glucose and 5.3 g/L tartaric acid, pH = 3.5) was used to establish the calibration curves. The standard solution was diluted into fifteen levels. Aroma standards of each level were extracted and analyzed in triplicate under the same conditions as the samples.

Table S4 Quantitative information for phenolic compounds by HPLC-QqQ-MS/MS.

NO	Retention time (min)	Compound	Ion (m/z)	Calibration curve			Linear range (mg/L)
				slope	intercept	R^2	
1	5.7	Malvidin-3-O-glucoside	493-331	4.00E-05	-37.441	0.9945	10-250
2	5.7	Malvidin-3-O-glucoside	493-331	7.00E-07	1.5203	0.9900	10-100
3	4.48	4-Hydroxybenzoic acid	137-93:3.4	0.0001	-6.0155	0.9931	1-50
4	2.7	Protocatechuic acid	153-109:2	6.00E-05	-0.4063	0.9927	1-10
5	4.4	Gentisic acid	153-109:3.3	0.0002	-1.0746	0.9967	1-12
6	9.6	4-Hydroxycinnamic acid	163-119:7.2	2.00E-06	0.1886	0.9913	0.1-20
7	12.3	3-Hydroxycinnamic acid	163-119:9.7	3.00E-05	-0.4208	0.9897	1-4
8	6.1	Vanillic acid	167-152:4.4	0.0022	0.1629	0.9867	0.1-10
9	1.5	Gallic acid	169-125	0.0002	-8.1	0.9736	1-100
10	6.4	Caffeic acid	179-135:4.3	3.00E-05	-6.7603	0.9875	10-80
11	11.9	Ferulic acid	193-134:9.2	0.0001	-1.5254	0.9946	1-40
12	12.5	Sinapic acid	223-149:9.9	0.0004	0.0703	0.9983	0.1-20
13	5.0	Catechin	289-123:3.2	0.0005	-5.9987	0.9874	1-125
14	8.4	Epicatechin	289-123:6	0.0004	-5.9383	0.9784	1-100
15	22.4	Quercetin	301-151:18.6	3.00E-05	-0.1961	0.9993	0.1-5
16	12.3	Dihydroquercetin	303-125:9.5	8.00E-05	-0.0922	0.9991	0.1-20
17	2.1	Gallocatechin	305-125:1.7	0.0007	5.5047	0.9865	1-160
18	4.4	Epigallocatechin	305-125:2.7	3.00E-06	-0.0686	0.9416	0.1-5
19	17.7	Myricetin	317-151:14.3	6.00E-05	-0.1415	0.9989	0.1-5
20	27.9	Chlorogentic acid	353-191:4	3.00E-05	-0.2521	0.9177	0.1-10
22	12.6	ECG	441-289:9.8	0.0002	-2.2685	0.9854	1-100
22	17.8	Kaempferol-galactoside	447-255:12.9	5.00E-05	0.2339	0.987	1-10
23	17.7	Quercetin-rhamnoside	447-300:14.5	3.00E-05	0.1314	0.9854	0.1-5
24	8.0	EGCG	457-169:5.03	6.00E-05	-1.9199	0.9932	1-125
25	14.6	Quercetin-galactoside	463-300:10.6	5.00E-05	-0.2203	0.9941	0.1-10
26	15.2	Quercetin-glucoside	463-300:12	5.00E-05	-1.0971	0.9983	1-40
27	14.8	Quercetin-glucuronide	477-301:11.8	7.00E-05	0.3239	0.9853	1-20
28	12.2	Myricetin-galactoside	479-316:8.6	3.00E-05	-0.1556	0.9891	1-10
29	5.7	Procyanin B1	577-407:2.5	0.0004	-1.9162	0.9937	1-50
30	6.8	Procyanin B2	577-407:4.4	0.0006	-1.4484	0.9937	1-40

Table S5 Preparation of reference standards for each sensory attribute*.

NO	Attributes	References
1	Blackberry	Dissolve 20 μL of blackberry essence in 20 ml of simulated wine solution.
2	Blueberry	Dissolve 20 μL of blueberry essence in 20 ml of simulated wine solution.
3	Strawberry	Dissolve 20 μL of strawberry essence in 20 ml of simulated wine solution.
4	Cherry	Dissolve 20 μL of cherry essence in 20 ml of simulated wine solution.
5	Raspberry	0.35 g of raspberry was treated with liquid nitrogen, and the crumbs were dissolved in 20 ml of simulated wine.
6	Mulberry	0.35 g of mulberry was treated with liquid nitrogen, and the crumbs were dissolved in 20 ml of simulated wine.
7	Banana	Dissolve 20 μL of banana essence in 20 ml of simulated wine solution.
8	Apple/Pear	Dissolve 20 μL of apple and pear essence in 20 ml of simulated wine solution.
9	Peach/Apricot	Dissolve 20 μL of blueberry essence in 20 ml of simulated wine solution.
10	Sophora flower	Dissolve 20 μL of sophora flower perfume in 20 ml of simulated wine solution.
11	Rose	Dissolve 20 μL of rose essence in 20 ml of simulated wine solution.
12	Violet	Le nez du vin
13	Dried/Preserved Fruit	Raisin
14	Herbaceous	0.35 g of herbaceous was treated with liquid nitrogen, and the crumbs were dissolved in 20 mL of simulated wine.
15	Toasted hazelnuts/toast	Le nez du vin
16	Honey	Dissolve 20 μL of honey essence in 20 ml of simulated wine solution.
17	Baked sweet potatoes	Dissolve 20 μL of baked sweet potato essence in 20 ml of simulated wine solution.
18	Oak	Oak chips
19	Tannin acid	Different concentrations of tannic acid aqueous solutions (0.5g/L, 1g/L, 2g/L, 2.5g/L, 4g/L, 5g/L)

* The model solution was prepared using12% ethanol/water solution, pH = 3.7.

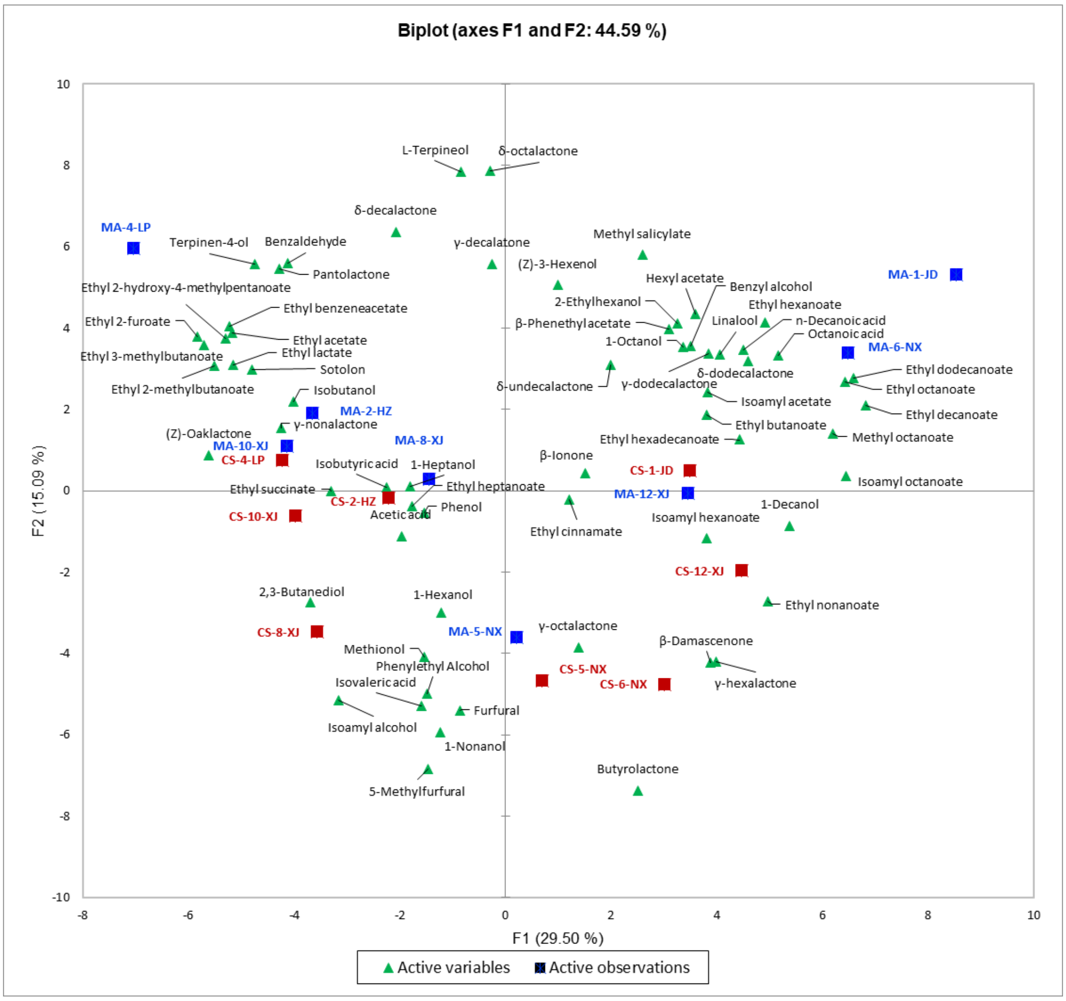

Figure S1. Principal component analysis of Cabernet Sauvignon and Marselan dry red wines from five regions in China based on aroma compounds. CS, Cabernet Sauvignon; MA, Marselan; LP, Loess Plateau; HZ, Huaizhuo; JD, Jiaodong; NX, Ningxia; XJ, Xinjiang.

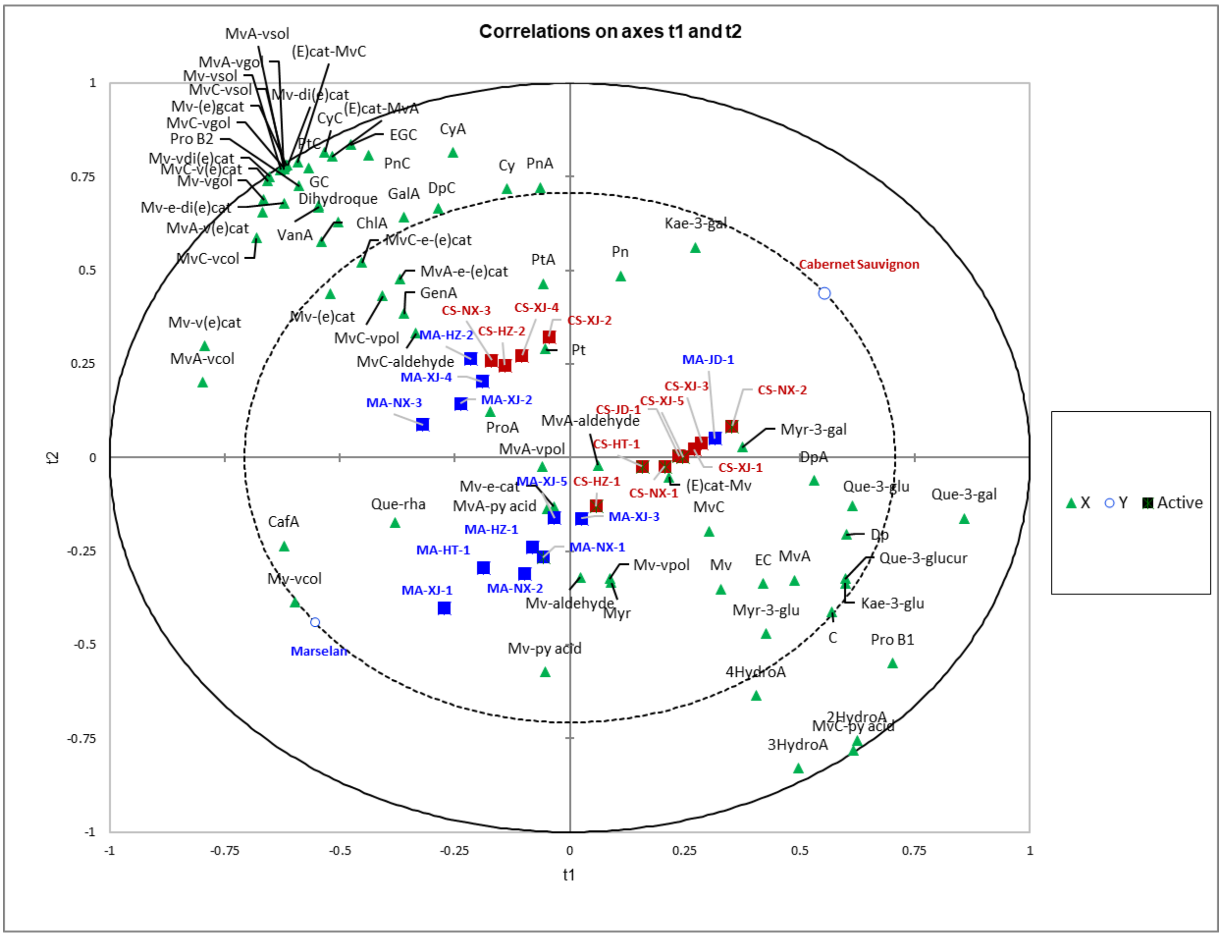

Figure S2. Partial least square-discriminant analysis of Cabernet Sauvignon and Marselan dry red wines from five regions in China based on phenolic compounds. Q^2=-0.291, R^2X=0.499, R^2Y=0.553. CS, Cabernet Sauvignon; MA, Marselan; , Loess Plateau; HZ, Huaizhuo; JD, Jiaodong; NX, Ningxia; XJ, Xinjiang.
